# Molecular markers associated with drug resistance in *Plasmodium falciparum* parasites in central Africa between 2016 and 2021

**DOI:** 10.3389/fpubh.2023.1239274

**Published:** 2023-08-30

**Authors:** Wenjie Xu, Xuan Zhang, Hualiang Chen, Jiaqi Zhang, Qiaoyi Lu, Wei Ruan, Xiaoxiao Wang

**Affiliations:** Zhejiang Provincial Center for Disease Control and Prevention, Zhejiang, China

**Keywords:** malaria, *Plasmodium falciparum*, molecular markers, parasites, Africa

## Abstract

**Objectives:**

The widespread occurrence of anti-malarial drug resistance threatens the current efforts to control malaria in African regions. Molecular marker surveillance helps to track the emergence and spread of drug-resistant malaria cases.

**Methods:**

A total of 237 *Plasmodium falciparum* infections imported from central Africa to Zhejiang Province, China, between 2016 and 2021, were investigated. Genomic DNA was extracted from blood samples of each patient and nested PCRs was used to detect molecular markers in *k13*, *Pfcrt*, and *Pfmdr1* genes. The spatial and temporal distributions of the molecular markers were analyzed.

**Results:**

A limited polymorphism of *k13* was observed, including two nonsynonymous (D464E and K503E) and five synonymous mutations. Wild-type CVMNK of *Pfcrt* predominated (78.5%), whereas 19.5% of the samples harbored the mutant haplotype, CVIET. The point mutation Y184F and the single mutant haplotype NF of *Pfmdr1* were the most frequently observed. The geographical distributions of the *Pfcrt* and *Pfmdr1* haplotypes displayed distinct patterns, with the mutant haplotype of *Pfcrt* more common in Gabon (53.9%) and Congo (50.0%), and wild haplotypes of *Pfmdr1* more frequently found in Cameroon, Angola, and Congo. The prevalence of wild-type CVMNK of *Pfcrt* increased from 68.5–74.6% in 2016–2017 to 81.8–87.5% in 2018–2021. The proportion of wild-type *Pfmdr1* also increased from 27.1% in 2016 to 38.5% in 2019.

**Conclusion:**

The geographical and temporal distribution of *k13*, *Pfcrt*, and *Pfmdr1* polymorphisms in *P. falciparum* parasites imported from central Africa between 2016 and 2021 are demonstrated. Our data provide updated evidence that can be used to adjust anti-malarial drug policies in central Africa and China.

## 1. Introduction

Malaria poses a significant threat to global public health. According to the World Malaria Report, 241 million clinical cases and 627,000 malaria-related deaths were reported worldwide in 2020 (1). *Plasmodium falciparum* is the most prevalent and virulent malaria species, causing approximately 90% of malaria-related deaths each year. The World Health Organization (WHO) African region accounts for 95% of malaria cases globally (1, 2). Malaria in Africa is also a continuing risk for international travelers, especially long-term migrant workers (2). Anti-malarials are the main weapon used against this potentially lethal infection (3). Despite significant progress in the fight against malaria worldwide, the spread of anti-malarial resistance threatens the current efforts to control malaria in Africa, as well as in countries where malaria cases have been imported (3, 4). China was certified as malaria-free by the WHO in June 2021 (5); however, the disease still poses a therapeutic challenge due to imported malaria cases.

Artemisinin resistance is primarily mediated by mutations in *P. falciparum* Kelch13 protein (K13), which involved in intracellular processes, such as endocytosis of hemoglobin (6). To date, a list of 10 validated *P. falciparum k13* markers and 11 candidate markers have been used to evaluate partial artemisinin (ART) resistance (7). It was thought that the validated *k13* markers were mainly limited to Southeast Asia until a validated marker, R561H, was identified in 7.4% of samples (19 of 257) between 2013 and 2015 in Rwanda, which had no genetic relationship with those previously reported in Southeast Asia. This finding has led to great concern given the disease burden in Africa (8). *Pfcrt*, which encodes the *P. falciparum* chloroquine resistance transporter, is a highly sensitive marker of chloroquine (CQ) treatment failure (9). The *Pfcrt* gene has a considerable polymorphism in codon position 72–76 that determines different haplotypes, including wild-type CVMNK, common mutation CVIET, SVMNT and mixed mutation type. The *P. falciparum* multidrug resistance protein transporter is a central node in a wide range of anti-malarial resistance through single nucleotide polymorphisms and/or copy numbers in *Pfmdr1* (10, 11). *Pfmdr1* is associated with parasite susceptibility to a variety of currently available anti-malarial drugs, including CQ, ART, lumefantrine, amodiaquine, mefloquine, and quinine (10). Most of them are important counterparts in first-line Artemisinin-based Combination Therapy (ACT); thus, *Pfmdr1* has become one of the pivotal factors in malaria resistance to artemisinin combination therapies (10, 11).

By subregion, central African countries are malaria endemic and accounted for nearly 25% of the estimated malaria cases in the WHO African region in 2020, which includes 10 countries of Congo DR, Angola, Cameroon, Equatorial Guinea, Gabon, Congo, Chad, Central African Republic, Sao Tome and Principe, and Burundi (1). A large number of malaria infections are imported from central Africa to China each year, particularly from Congo, Angola, Cameroon, etc. (12, 13). This poses a great risk to China in maintaining a malaria-free status, as imported patients with anti-malarial drug resistance might result in failure or delay in parasite clearance (14, 15). Therefore, it is necessary to follow the epidemiological patterns of imported malaria infections that are resistant to malaria drugs. Herein, we examined the prevalence of the molecular markers of *k13, pfcrt*, and *pfmdr1*, and their polymorphisms in imported *P. falciparum* malaria cases from central Africa between 2016 and 2021 in Zhejiang province, China. This study aimed to provide useful information for a deeper understanding of the status and spectrum of drug resistance of the *P. falciparum* parasite in central Africa by examining the molecular epidemiology traits of imported cases, and to gain information that can be used to provide guidance on medication in clinical practice and drug administration.

## 2. Materials and methods

### 2.1. Study site

Zhejiang Province, in eastern China, was certified as malaria-free in 2018. All the malaria patients were imported from malaria endemic countries since 2011. Specifically, a total of 298 malaria cases were imported from central Africa between 2016 and 2021, among which the majority (79.5%, 237/298) were infected with *P. falciparum,* followed by *Plasmodium ovale* (16.4%, 49/298), *Plasmodium malariae* (3.0%, 9/298), and *Plasmodium vivax* (1.0%, 3/298).

### 2.2. Diagnosis of malaria cases

According to national guidelines on malaria diagnosis, each case with malaria-related symptoms was sampled using venous blood and sent to the laboratory for microscopy or rapid diagnostic tests (RDTs). Positive results were reported by hospitals or clinics to the local Center for Disease Control and Prevention (CDC) as well as the Zhejiang Provincial CDC. The local CDC conducted epidemiological investigations in order to trace parasite origins. The Zhejiang Provincial CDC analyzed and confirmed the species via thick and thin blood smears as well as PCR for each patient.

### 2.3. Sample collection and DNA extraction

In total, 237 *P. falciparum* parasites were identified. Approximately 1 mL of venous blood was collected from each patient prior to anti-malarial treatment. All blood samples were stored at −80°C until use. Genomic DNA was extracted using the QIAamp DNA Mini Kit (QIAGEN, Hilden, Germany) according to the manufacturer’s instructions.

### 2.4. DNA amplification and sequencing

Molecular markers in *k13*, *Pfcrt*, and *Pfmdr1* genes, which are associated with the drug resistance of *P. falciparum* parasites, were detected via nested PCR, as previously described (16–18). An 849-bp amplification product of *k13* was obtained and sequences covering amino acid position 72–76 of *Pfcrt* and 86 and 184 of *Pfmdr1* were amplified. The primers used for the genotyping assay are listed in Supplementary Table S1 (18). The cycling conditions for *k13* and *Pfcrt* genes have been published previously (18). Amplification conditions in both PCR rounds for *Pfmdr1* were as follows: one cycle at 95°C for 5 min, followed by 30 cycles at 95°C for 1 min, 54°C for 90 s, and 72°C for 1 min, and an extension cycle at 72°C for 10 min. The PCR products were sequenced using Sanger sequencing (Sangon Biotech Co. Ltd., Shanghai, China). Both positive controls and negative controls were set during each round of nested PCR. Further, about 5% samples were randomly selected for duplicate detection to confirm the results of nested PCR.

### 2.5. Data analysis

The database was constructed using Microsoft Excel 2017. Mega version 7.0.26^1^ was used to align amplicon sequences with reference sequences retrieved from the NCBI database. Accession numbers for the reference sequences were: *k13* (XM_001350122.1), *Pfcrt* (NC_004328.3), and *Pfmdr1* (X56851.1). To analyze the geographical and temporal distribution of haplotypes of *Pfcrt* and *Pfmdr1*, the samples were classified into wild and mutant haplotype groups according to sequencing. The *χ^2^* test was used to compare the geographical and temporal differences. Statistical significance was set at a value of *p* < 0.05.

## 3. Results

### 3.1. General information

**Figure 1:**
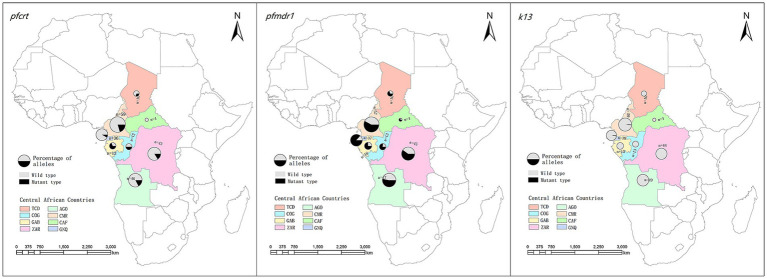
Geographical distribution of imported *Plasmodium falciparum* parasites from central Africa and their patterns of haplotypes for *Pfcrt*, *Pfmdr1* and *k13*. TCD, Chad; AGO, Angola; COG, Congo; CMR, Cameroon; GAB, Gabon; CAF, Central African Republic; COD, Congo DR; GNQ, Equatorial Guinea.

Of the 10 countries in central Africa, malaria patient information was imported from eight countries (Figure 1; Supplementary Table S2). Among these countries, Cameroon contributed the largest number of *P. falciparum* cases (26.2%, 62/237), followed by Angola (20.7%, 49/237) and Congo (19.0%, 45/237), whereas there were no infections imported from Burundi, Sao Tome, or Principe. We found that the number of *P. falciparum* cases from central African countries declined substantially over time, from 72 cases in 2016 to 13 cases in 2021.

### 3.2. *k13* propeller polymorphisms

Of the 237 samples, 232 were successfully amplified and sequenced. A limited polymorphism in *k13* was observed in this study. Briefly, none of the candidate or validated mutations associated with resistance were detected in the samples that were analyzed. Nevertheless, seven samples (3.1%, 7/232) presented point mutations, including two (0.9%, 2/232) non-synonymous mutations (D464E and K503E) and five (2.1%, 5/232) synonymous mutations (F442F, R471R, T535T, V589V, and G690G; Table 1). The D464E mutation was detected in one sample from Equatorial Guinea in 2018, whereas K503E was identified in a Cameroon sample in 2018.

**Table 1:** Polymorphisms observed in *k13* of imported *Plasmodium falciparum* parasites from central Africa (*n* = 232).

Codon position	Amino acid reference	Nucleotide reference	Amino acid mutation	Nucleotide mutation	Location and year (No.)
442	F	TTC	F	TTT	Cameroon, 2017 (1)
464	D	GAT	E	GAA	Equatorial Guinea, 2018 (1)
471	R	CGT	R	CGC	Gabon, 2017 (1)
503	K	AAG	E	GAG	Cameroon, 2018 (1)
535	T	ACG	T	ACA	Chad, 2019 (1)
589	V	GTC	V	GTG	Cameroon, 2019 (1)
690	G	GGC	G	GGA	Cameroon, 2018 (1)

### 3.3. *Pfcrt* polymorphisms and its geographical distribution

Of the 237 samples, 223 were successfully genotyped across *Pfcrt* codons 72–76. Wild-type *Pfcrt* alleles (CVMNK), mutant alleles (CVIET), and mixed-type alleles (CVM/I N/E/D/K K/T) were observed. The most frequent haplotype was the wild-type CVMNK (78.5%, 175/223), whereas 19.5% (39/223) of the samples harbored the mutant haplotype. Additionally, the mixed type was found in nine samples (4.0%).

Regarding the spatial distribution of *Pfcrt* polymorphisms, the percentage of wild, mutant, and mixed types varied between countries. Mutant haplotypes were more common in patients from Gabon (53.8%) and Congo (50.0%), whereas wild-type CVMNK was predominant in Equatorial Guinea (91.7%), Congo DR (83.7%), Angola (82.6%), and Cameroon (79.7%). Although the number of cases returning from the Central Africa Republic was limited, all parasites were wild-type (Table 2). When looking at the mutant type and mixed type together, no statistically significant difference in the prevalence of wild and mutant haplotypes was revealed among the countries (*χ^2^* = 29.722, *p < 0.001*; Figure 1; Supplementary Table S3).

**Table 2:** Polymorphisms observed in *Pfcrt* of imported *Plasmodium falciparum* parasites from Central Africa (*n* = 223).

Country	No. of samples	Haplotype (%)		
		Wild type	Mutant type	Mixed type
		CVMNK	CVIET	CVM/I N/E/D/K K/T
Cameroon	59	47 (79.7)	9 (15.3)	3 (5.1)
Angola	46	38 (82.6)	6 (13.0)	2 (4.3)
Congo DR	43	36 (83.7)	6 (14.0)	1 (2.3)
Equatorial Guinea	36	33 (91.7)	3 (8.3)	0
Gabon	13	4 (30.8)	7 (53.8)	2 (15.4)
Congo	12	6 (50.0)	6 (50.0)	0
Chad	10	7 (70.0)	2 (20.0)	1 (10.0)
Central African Republic	4	4 (100.0)	0	0
Total	223	175 (78.5)	39 (19.5)	9 (4.0)

### 3.4. *Pfmdr1* polymorphisms and its geographical distribution

Of the 237 *P. falciparum* parasites from central Africa described above, 229 were successfully sequenced. Generally, the prevalence of the point mutations, N86Y and Y184F, was 14.4% (33/229) and 61.1% (140/229), respectively (Table 3). However, the percentage of single mutants varied significantly across countries. Chad had the largest proportion of N86Y (30.0%), followed by the Central African Republic (25.0%), and Equatorial Guinea (24.3%). Y184F was frequently observed in Equatorial Guinea (86.5%), the Central African Republic (75.0%), and Chad (70.0%). Furthermore, uncommon I107V and G102G mutations, resulting from a nucleotide mutation (T to C at position 306), were recorded. I107V was found in an isolate from Angola in 2016, whereas G102G was present in two samples from Cameroon and two samples from Chad.

**Table 3:** Polymorphisms observed in *Pfmdr1* of imported *Plasmodium falciparum* parasites from central Africa (*n* = 229).

Country	No. of samples	SNPs (%)		Haplotypes (%)			
		N86Y	Y184F	NY	YY	NF	YF
Cameroon^*^	57	9 (15.8)	31 (54.4)	24 (42.1)	2 (3.5)	24 (42.1)	7 (12.3)
Angola^△^	47	3 (6.4)	23 (48.9)	22 (46.8)	0 (0)	22 (46.8)	3 (6.4)
Congo DR	45	4 (8.9)	24 (53.3)	18 (40.0)	1 (2.2)	24 (53.3)	2 (4.4)
Equatorial Guinea	37	9 (24.3)	32 (86.5)	5 (13.5)	0 (0)	22 (59.5)	10 (27.0)
Gabon	16	3 (18.8)	11 (68.8)	4 (25.0)	(6.3)	9 (56.3)	2 (12.5)
Congo	13	1 (7.7)	9 (69.2)	3 (23.1)	1 (7.7)	9 (69.2)	0 (0)
Chad^*^	10	3 (30.0)	7 (70.0)	3 (30.0)	0 (0)	4 (40.0)	3 (30.0)
Central African Republic	4	1 (25.0)	3 (75.0)	1 (25.0)	0 (0)	2 (50.0)	1 (25.0)
Total	229	33 (14.4)	140 (61.1)	80 (34.9)	5 (2.2)	116 (50.7)	28 (12.2)

* Two samples harboring G102G.

^△^One sample harboring I107V.

Four haplotypes were identified, including wild-type NY, single mutations YY and NF, and double mutation YF. In total, single-mutant type NF prevailed in 50.7% (116/229) of cases. Wild-type NY was also common and observed in 34.9% (80/229) of the samples, with higher frequencies in Cameroon, Angola, and Congo.

Single mutant NF predominated in Congo (69.2%), Equatorial Guinea (59.5%), Congo (53.3%), and Gabon (56.3%). Furthermore, Chad and Equatorial Guinea had a high prevalence of the double mutation YF (30.0 and 27.0%, respectively).

### 3.5. Temporal distribution for haplotypes of *Pfcrt* and *Pfmdr1*

The prevalence of wild-type CVMNK of *Pfcrt* increased from 68.5–74.6% in 2016–2017 to 81.8–87.5% in 2018–2021, while the frequency of the mutant type declined, although no statistically significant difference was found (*χ^2^* = 7.596, *p* = *0.18*). Regarding *Pfmdr1*, the proportion of wild-type also increased from 27.1% in 2016 to 38.5% in 2019, whereas the percentage of mutant type decreased from 72.9 to 61.5% (*χ^2^* = 5.027, *p* = *0.413*). Due to the limited sample size in 2020 and 2021, no regular pattern of *Pfmdr1* was observed in the period between 2020 and 2021 (Table 4).

**Table 4:** Temporal distribution of haplotypes of *Pfcrt* and *Pfmdr1.*

Year	*Pfcrt*				*Pfmdr1*			
	Wild type (CVMNK)	Mutant type^*^ (CVIET)	*χ^2^*	*p* value	Wild type (NY)	Mutant type (YY, NF, and YF)	*χ^2^*	*p* value
2016	50 (74.6)	17 (25.4)	7.596	0.18	19 (27.1)	51 (72.9)	5.027	0.413
2017	37 (68.5)	17 (31.5)			21 (37.5)	35 (62.5)		
2018	38 (86.4)	6 (13.6)			16 (35.6)	29 (64.4)		
2019	35 (87.5)	5 (12.5)			15 (38.5)	24 (61.5)		
2020	6 (85.7)	1 (14.3)			6 (60.0)	4 (40.0)		
2021	9 (81.8)	2 (18.2)			3 (33.3)	6 (66.7)		
Total	175 (78.5)	48 (21.5)			80 (34.9)	149 (65.1)		

* Mixed type included.

## 4. Discussion

It is essential that anti-malarial drugs remain effective in saving the lives of millions of people infected with malaria. Monitoring drug resistance is important because early detection enables a timely action in order to prevent its spread and to mitigate any impact on global health. Most of the global burden of *P. falciparum* is in the WHO African region. In 2020, it was estimated that there were more than 54 million infections and almost 140,100 deaths in central Africa and approximately 191 million people living in the 10 countries of central Africa are at high risk of malaria (1). Here, we investigated the genetic mutations of *k13*, *Pfcrt*, and *Pfmdr1* associated with anti-malarial resistance in malaria cases imported from central Africa to Zhejiang province, China. Our purpose was to better understand the status and spectrum of drug resistance of parasites in central Africa using molecular epidemiology and to provide evidence for drug policy updates in the study area.

The present observational study illustrated a low prevalence of nonsynonymous mutations of *k13* (0.9%, 2/232) in the samples analyzed, indicating limited *k13* polymorphism in central African countries. This finding is comparable with the available data (19–22). A review by the WHO indicated that most of the samples in central African countries were *k13* wild type (98.6%) (23). In summary, 61 mutations were identified, of which five (M476I, P574L, P553L, R539T, and R561H) were validated molecular markers and one (C469F) was a candidate molecular marker associated with partial ART resistance (23). Notably, neither validated markers nor candidate markers were identified in our study. Regarding the two non-synonymous mutations (D464E and K503E) revealed in this study, there is still no evidence confirming their roles in mutant *k13*-mediated ART resistance. In addition to the validated and candidate molecules, the rare frequency of some nonsynonymous mutations, such as D464E, K503E, and I448L in Cameroon, V454A in Gabon, and V534A in Congo, described in previous studies, underlines the abundant polymorphism of *k13* in central Africa (24). At the country level, Cameroon presented more *k13* polymorphisms than any other central African country, with one sample possessing nonsynonymous mutations, and three samples possessing synonymous mutations. Although this difference may be due to more participants being enrolled from Cameroon in the current study, continued monitoring of the trend of molecular determinants is warranted, given the genetically independent rise of R561H in African isolates since 2014 (8).

It was demonstrated that the mutant haplotype CVIET in *Pfcrt* conferred a high level of resistance and was the most common in Africa (25). Our data demonstrated a low proportion (19.5%, 39/223) of mutant type CVIET in *Pfcrt* at positions 72–76, indicating the return of CQ-sensitive *P. falciparum* parasites in central Africa. This was consistent with previous studies, which revealed a prevalence of more than 90% prior to CQ withdrawal in 2002, which declined to 73.2% 8 years after CQ replacement in Congo (26, 27), as well as the reemergence of *P. falciparum* isolates with wild-type *Pfcrt* in Cameroon, Angola, and Equatorial Guinea (22, 28, 29). Based on our findings, it is suggested that the selection of *Pfcrt* was relieved in most central African countries after long-term ACT use. However, Gabon was underscored in the present study because of its low prevalence of wild-type CVMNK (30.8%) compared with other countries from central Africa. Although the current study was limited by the small size of Gabon, the results were in agreement with those of another study, which showed a low wild-type proportion of 16.5–29.4% in Gabon (30).

Combinations of SNPs in codons N86Y, Y184F, and D1246Y of *Pfmdr1* confer reduced sensitivity to different drugs. Parasites with wild N86 was estimated 4.7 times more likely to recrudesce after Artemether–lumefantrine treatment than those with the N86Y genotype (31, 32). Concerning the polymorphism of *Pfmdr1*, we found that Y184F was the predominant mutation with a prevalence of 61.1%, which was much higher than those that identified in central Africa, as well as east and west Africa, in other published studies (33, 34). Furthermore, more than half of the samples from central Africa in the current study harbored a single mutant haplotype NF, suggesting that NF was more common in central Africa than previously thought (35). In addition, the frequency of haplotypes of *Pfmdr1* varied greatly between countries. The single mutant haplotype NF was more frequently observed in Congo (69.2%), Gabon (56.3%), and Equatorial Guinea (59.5%) when compared to other countries, indicating the diversity of drug pressure and transmission intensity among African countries or regions.

Regarding the temporal distribution of haplotypes of *Pfcrt* and *Pfmdr1,* our data indicated a return of sensitive *Pfcrt* and *Pfmdr1*. Specifically, there was an increase in the prevalence of wild-type CVMNK from 68.5–74.6% in 2016–2017 to 81.8–87.5% in 2018–2021. For *Pfmdr1*, an increasing proportion of wild-type NY and declining frequency of mutant type were demonstrated between 2016 and 2019. The moderate rise of wild-type *Pfcrt* and *Pfmdr1* in the present study indicated the relief of drug pressure due to the cessation of CQ use in central African countries (27, 28). Therapeutic efficacy studies in central Africa are recommended for tracking clinical and parasitological outcomes (36).

This study had some limitations. First, there was a small sample size in several countries, especially the northern countries of central Africa, which limited further interpretations of the prevalence of molecular markers. However, the geographic distribution of the parasites in the present study was similar to that of malaria cases in central African countries. Congo, Angola, and Cameroon account for approximately 80% of the estimated infections (1), and the imported *P. falciparum* cases in our study were mainly from these three countries. Second, no data on the success or clinical failure of the treatments were collected, as the current study focused on the molecular markers associated with drug resistance in *P. falciparum* parasites.

## 5. Conclusion

In this study, we demonstrated the geographical and temporal distribution of *k13* propeller, *Pfcrt* and *Pfmdr1* polymorphisms in *P. falciparum* parasites imported from central Africa between 2016 and 2021. A limited *k13* propeller polymorphism was identified. The relief of antimalarial drug pressure in central Africa was indicated by predominant wild type CVMNK of *Pfcrt* and the single mutant of *Pfmdr1* in most countries, as well as their increasing trend during the study period between 2016 and 2021. The profiles of haplotypes of *Pfcrt* and *Pfmdr1* displayed varied spatial patterns among countries. Especially, Gabon and Equatorial Guinea required further strategy and measures as their higher proportion of mutant/mixed type of *Pfcrt* or *Pfmdr1*, comparing with other countries. Our data provides updated evidence that can be used to adjust national anti-malarial drug policies in central Africa and China.

## Data availability statement

The original contributions presented in the study are included in the article/Supplementary material, further inquiries can be directed to the corresponding authors.

## Ethics statement

The studies involving humans were approved by Ethical Review Committee of Zhejiang Center for Disease Control and Prevention. The studies were conducted in accordance with the local legislation and institutional requirements. The ethics committee/institutional review board waived the requirement of written informed consent for participation from the participants or the participants’ legal guardians/next of kin because Malaria is the notificable infectious in China, and the epidemiological investigation of notificable infectious cases are conducted in accordance with the Infectious Disease Prevention and Control Law.

## Author contributions

XW and WR conceived this study. HC, JZ, and QL carried out the molecular studies. XZ analyzed the data. WX and XW drafted the manuscript. All authors contributed to the article and approved the submitted version.

## Funding

This work was funded by grants from the Basic public welfare research project of Zhejiang Province (Grant No. LGF22H260006) and Medical Research Program of Zhejiang Province (Grant No. 2021KY121 and 2022KY723).

## Conflict of interest

The authors declare that the research was conducted in the absence of any commercial or financial relationships that could be construed as a potential conflict of interest.

## Publisher’s note

All claims expressed in this article are solely those of the authors and do not necessarily represent those of their affiliated organizations, or those of the publisher, the editors and the reviewers. Any product that may be evaluated in this article, or claim that may be made by its manufacturer, is not guaranteed or endorsed by the publisher.

## Footnotes

^1^https://www.megasoftware.net/
