# Supplementary material for: Molecular markers associated with drug resistance in Plasmodium falciparum parasites in central Africa between 2016 and 2021
Source: Front Public Health. 2023 Aug 30;11:1239274. doi: 10.3389/fpubh.2023.1239274 (PMC10499197; doi:10.3389/fpubh.2023.1239274)
Supplement: Supplementary file 1 [file Data_Sheet_1.DOCX]

TABLE S1 Primers for *k13*, *Pfcrt* and *Pfmdr1* genotyping assay

| Gene | Round | Primer name | Sequence (5’→3’) |
| --- | --- | --- | --- |
| *k13* | Primary | K13-1-F | CGGAGTGACCAAATCTGGGA |
|  |  | K13-1-R | GGGAATCTGGTGGTAACAGC |
|  | Secondary | K13-2-F | GCCAAGCTGCCATTCATTTG |
|  |  | K13-2-R | GCCTTGTTGAAAGAAGCAGA |
| *Pfcrt* | Primary | *Pfcrt*-1-F | CCCTTGTCGACCTTAACAGATG |
|  |  | *Pfcrt*-1-R | AAAATGACTGAACAGGCATCTAAC |
|  | Secondary | *Pfcrt*-2-F | TCTTGGTAAATGTGCTCATGTG |
|  |  | *Pfcrt*-2-R | AAAGTTGTGAGTTTCGGATGTT |
| *Pfmdr1* | Primary | *Pfmdr1*-1-F | TTAAATGTTTACCTGCACAACATAGAAAATT |
|  |  | *Pfmdr1*-1-R | CTCCACAATAACTTGCAACAGTTCTTA |
|  | Secondary | *Pfmdr1*-2-F | TGTATGTGCTGTATTATCAGGA |
|  |  | *Pfmdr1*-2-R | CTCTTCTATAATGGACATGGTA |

TABLE S2 *P. falciparum* cases imported from central Africa to Zhejiang Province between 2016 and 2021

| Country | No. of *P. falciparum* cases by Year | | | | | | Total |
| --- | --- | --- | --- | --- | --- | --- | --- |
|  | 2016 | 2017 | 2018 | 2019 | 2020 | 2021 |  |
| Cameroon | 15 | 13 | 12 | 9 | 4 | 9 | 62 |
| Angola | 24 | 10 | 5 | 6 | 3 | 1 | 49 |
| Congo DR | 13 | 10 | 9 | 9 | 2 | 2 | 45 |
| Equatorial Guinea | 11 | 9 | 13 | 4 | 0 | 1 | 38 |
| Gabon | 2 | 8 | 4 | 1 | 1 | 0 | 16 |
| Congo | 4 | 3 | 1 | 5 | 0 | 0 | 13 |
| Chad | 1 | 2 | 1 | 6 | 0 | 0 | 10 |
| Central African Republic | 2 | 2 | 0 | 0 | 0 | 0 | 4 |
| Total | 72 | 57 | 45 | 40 | 10 | 13 | 237 |

Table S3 The geographic distribution of haplotypes of *Pfcrt* and *Pfmdr1*

| Country | *Pfcrt* | | | |  | *Pfmdr1* | | | |
| --- | --- | --- | --- | --- | --- | --- | --- | --- | --- |
|  | Wild type  (CVMNK) | Mutant type*  (CVIET) | *χ^2^* | *P* value |  | Wild type  (NY) | Mutant type  (YY, NF, YF) | *χ^2^* | *P* value |
| Cameroon | 47 (79.7) | 12 (20.3) | 29.722 | <0.001 |  | 24 (42.1) | 33 (57.9) | 13.962 | 0.052 |
| Angola | 38 (82.6) | 8 (17.4) |  |  |  | 22 (46.8) | 25 (53.2) |  |  |
| Congo DR | 36 (83.7) | 7 (16.3) |  |  |  | 18 (40) | 27 (60) |  |  |
| Equatorial Guinea | 33 (91.7) | 3 (8.3) |  |  |  | 5 (13.5) | 32 (86.5) |  |  |
| Chad | 7 (70) | 3 (30) |  |  |  | 3 (30) | 7 (70) |  |  |
| Congo | 6 (50) | 6 (50) |  |  |  | 3 (23.1) | 10 (76.9) |  |  |
| Gabon | 4 (30.8) | 9 (69.2) |  |  |  | 4 (25) | 12 (75) |  |  |
| Central African Republic | 4 (100) | 0 (0) |  |  |  | 1 (25) | 3 (75) |  |  |
| Total | 175 (78.5) | 48 (21.5) |  |  |  | 80 (34.9) | 149 (65.1) |  |  |

*: Mixed type included.

* Mixed type included.

Figure S1 Temporal distribution of haplotypes in *Pfcrt* of *P. falciparum* cases

imported from central Africa countries between 2016 and 2021

Figure 2 Temporal distribution of haplotypes in *Pfmdr1* of *P. falciparum* cases

imported from central Africa countries between 2016 and 2021
